# Supplementary figures and images for: Evolution of two metabolic genes involved in nucleotide and amino acid metabolism in Pseudomonas aeruginosa
Source: PLoS One. 2024 Dec 17;19(12):e0315931. doi: 10.1371/journal.pone.0315931 (PMC11651626; doi:10.1371/journal.pone.0315931)

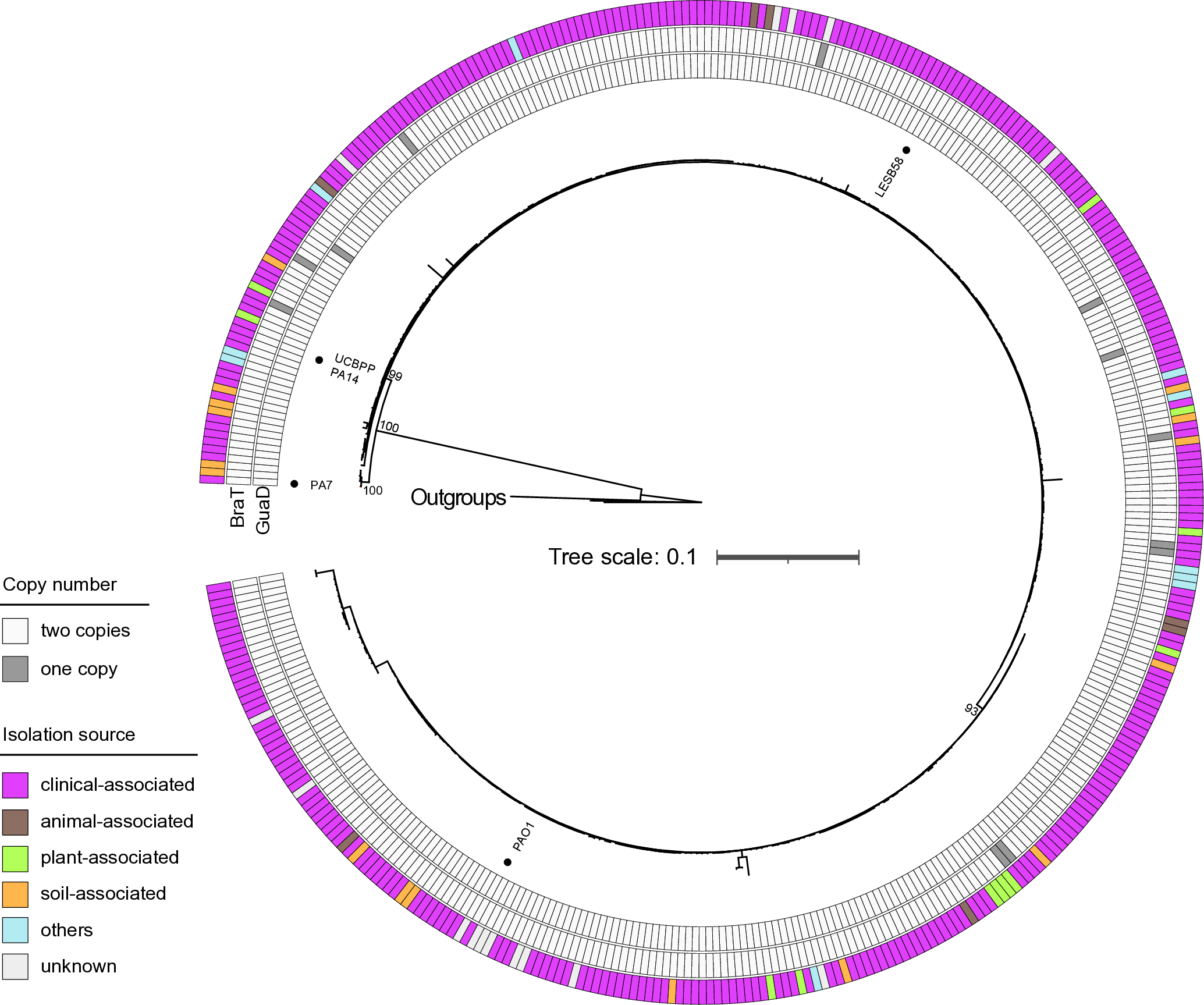

Supplement: S1 Fig — The phylogeny of 391 P. aeruginosa strains was built using IQ-TREE based on the concatenation of the 120 marker proteins with 1000 bootstrap replicates. The scale bar represents 0.1 substitutions per site and the bootstrap values of major clades are shown at the nodes. The copy numbers of guanine deaminase (GuaD) or transporters specific to BCAA (BraT) are indicated by the inner strips, with white for two copies and grey for one copy. The isolation sources are indicated by the outer strips. Purple, brown, green, orange and light blue strips represent clinical-associated, animal-associated, plant-associated, soil-associated and other samples, respectively. Four representative strains are marked with black dots, including PAO1, LESB58, UCBPP_PA14 and PA7. (TIF) [file pone.0315931.s001.tif]

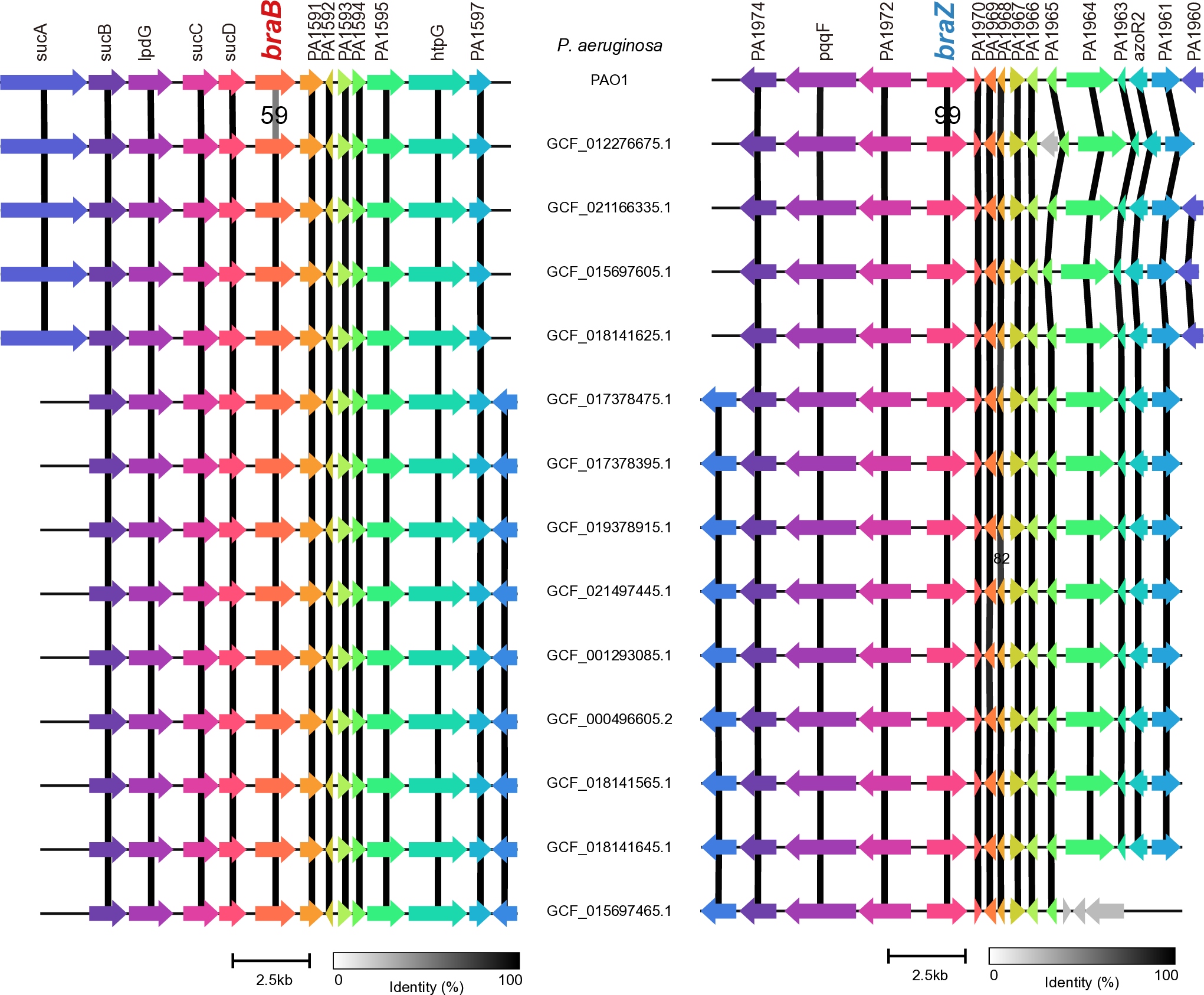

Supplement: S2 Fig — Colorful arrows and dark shading indicate the gene direction and nucleotide sequence identity of conserved regions. braZ_copy 1 in the sub-clade 1 of Fig 1A (right) shared the same genetic organization of genomic regions flanking braB, but with low identity of 59% between braZ_copy 1 and braB (left). braZ_copy 2 in the sub-clade 2 of Fig 1A (right) shared the same genetic organization of genomic regions flanking braZ, with high identity of 99% between braZ_copy 2 and braZ (right). The nucleotide sequence identity less than 90% are indicated. The scale bar indicates the length of 2.5kb nucleotides. (TIF) [file pone.0315931.s002.tif]

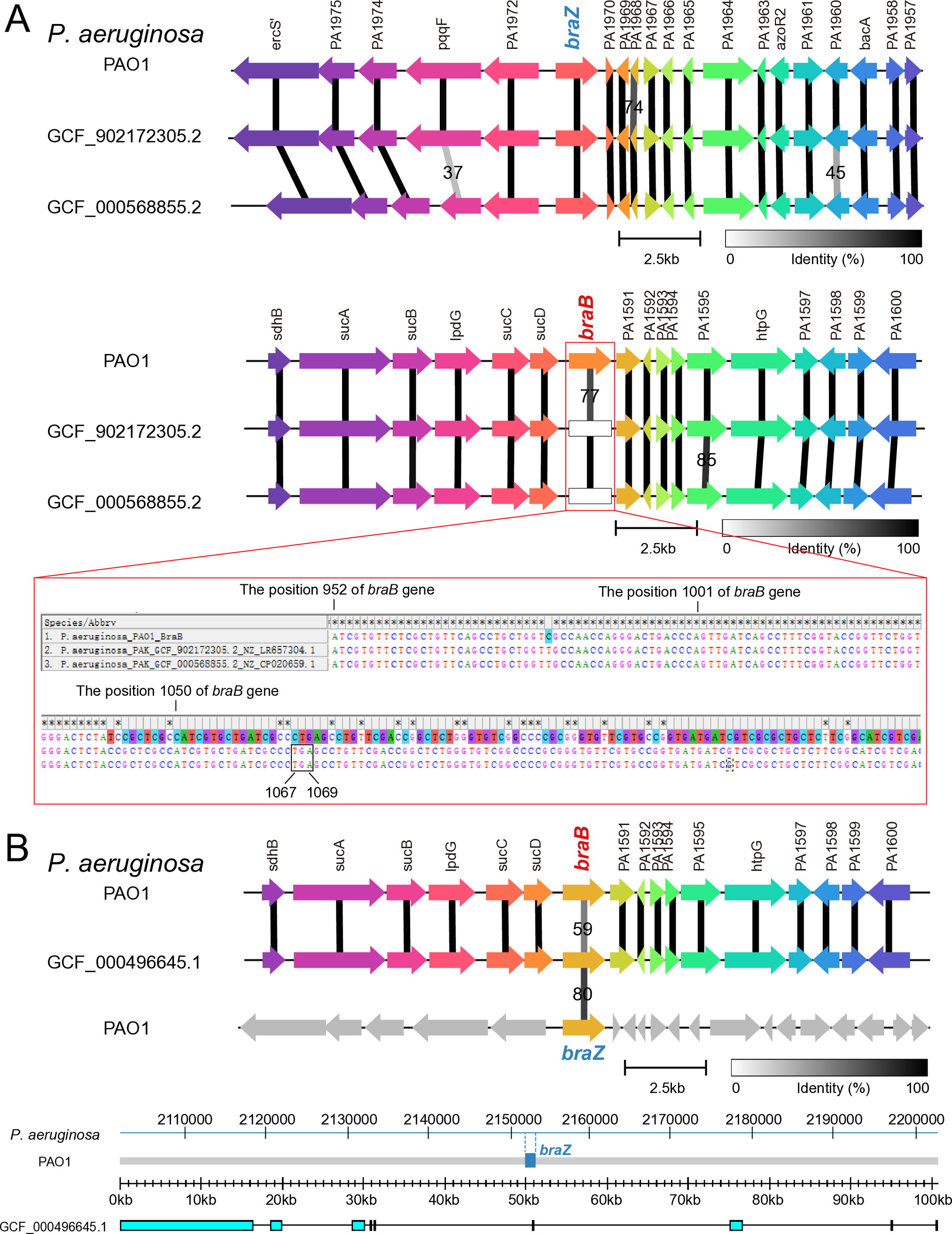

Supplement: S3 Fig — Colorful arrows and dark shading indicate the gene direction and nucleotide sequence identity of conserved regions. (A) The open reading frame was frameshifted at the position 1041, and stop codon was found at the position 1067–1069 in P. aeruginosa PAK GCF_902172305.2 and P. aeruginosa PAK GCF_000568855.2, resulting in the deleted protein sequences of braB. (B) For P. aeruginosa PA1R GCF_000496645.1, braZ_copy in the sub-clade 1 of Fig 1A (right) sharing the same genetic organization of genomic regions flanking braB, but with low identity of 59% between braZ_copy and braB. About 80kb of genomic region flanking the original position of braZ was lost. (TIF) [file pone.0315931.s003.tif]

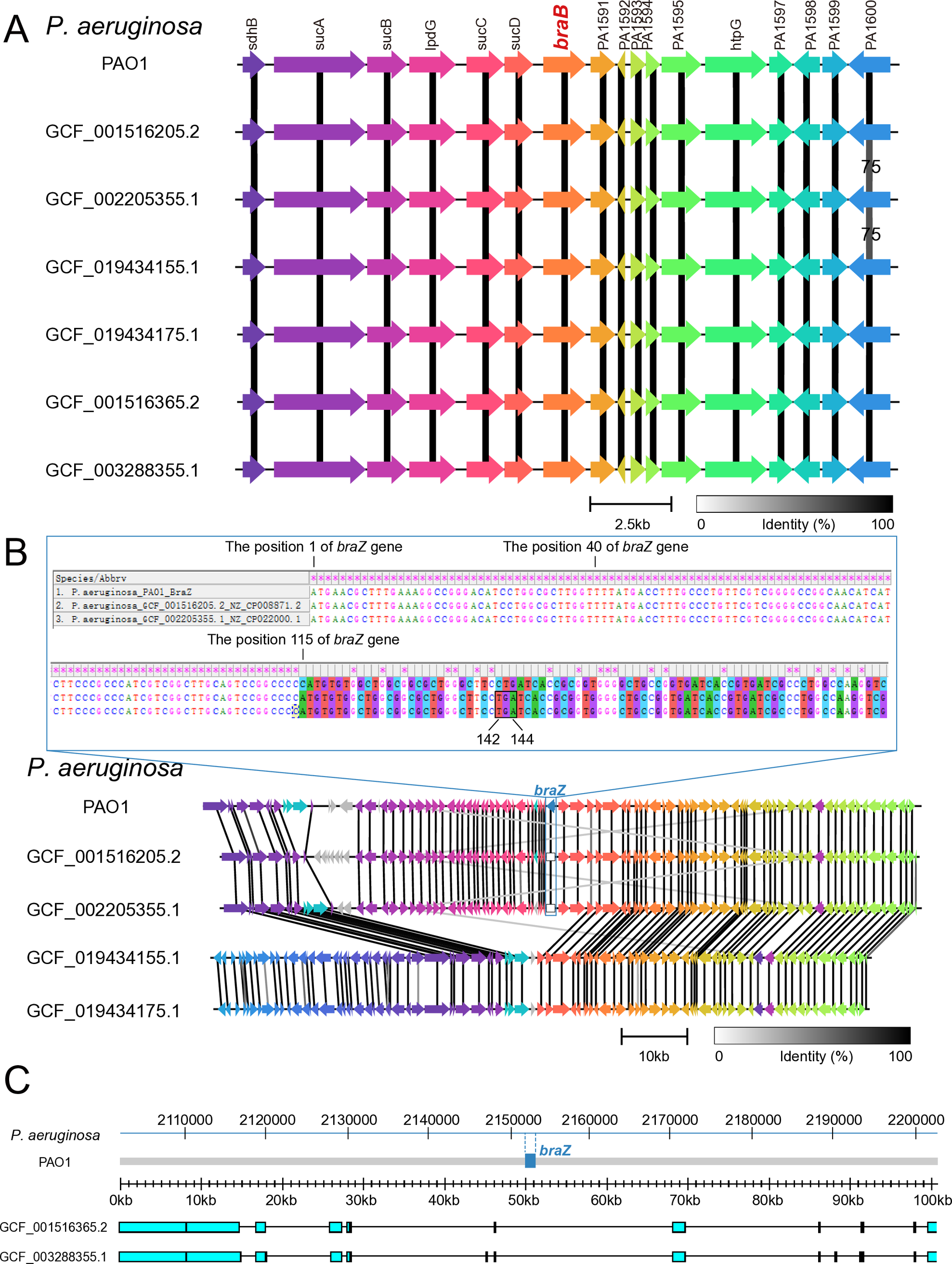

Supplement: S4 Fig — Colorful arrows and dark shading indicate the gene direction and nucleotide sequence identity of conserved regions. (A) All six strains shared the same genetic organization of genomic regions flanking braB. (B) The open frame was frameshifted at the position 115, and stop codon was found at the position 142–144 in P. aeruginosa GCF_001516205.2 and P. aeruginosa GCF_002205355.1, resulting in the deleted protein sequences of braZ. For P. aeruginosa GCF_019434155.1 and P. aeruginosa GCF_019434175.1, about 41kb genomic region was lost in each strain. And the deleted genomic region ranges from 2116000 to 2157701 referred to P. aeruginosa PAO1, consisting of braZ with 2151755–2153068. (C) For P. aeruginosa GCF_001516365.2 and P. aeruginosa GCF_003288355.1, about 81kb genomic region was lost in each strain. And the deleted genomic region ranges from 2120000 to 2201000 referred to P. aeruginosa PAO1, consisting of braZ with 2151755–2153068. (TIF) [file pone.0315931.s004.tif]

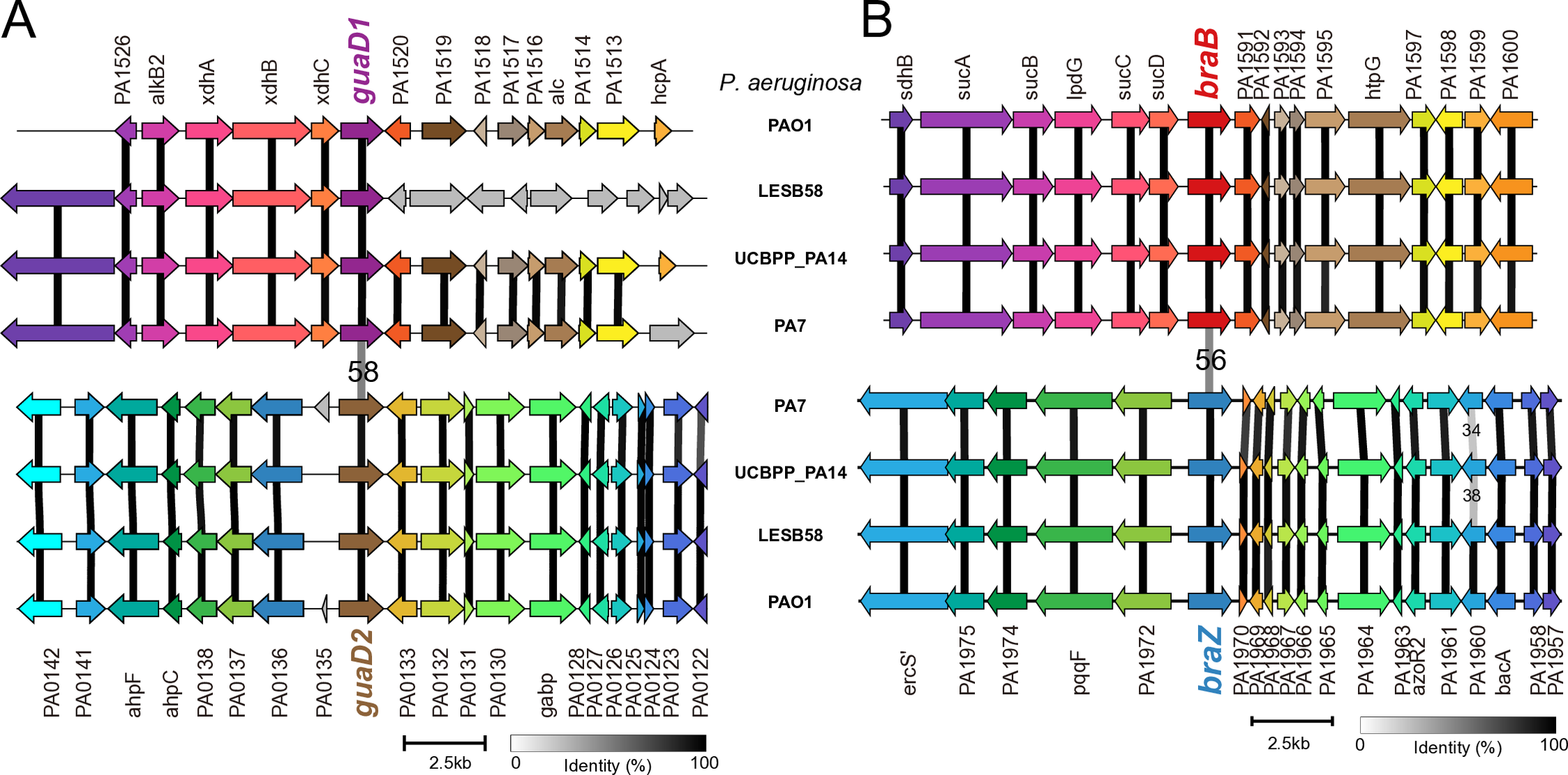

Supplement: S5 Fig — Colorful arrows and dark shading indicate the gene direction and nucleotide sequence identity of conserved regions. No dark shading was found between genomic regions flanking two copies, except between braB and braZ, as well as guaD1 and guaD2. The nucleotide sequence identity less than 90% are indicated. The scale bar indicates the length of 2.5kb nucleotides. (TIF) [file pone.0315931.s005.tif]

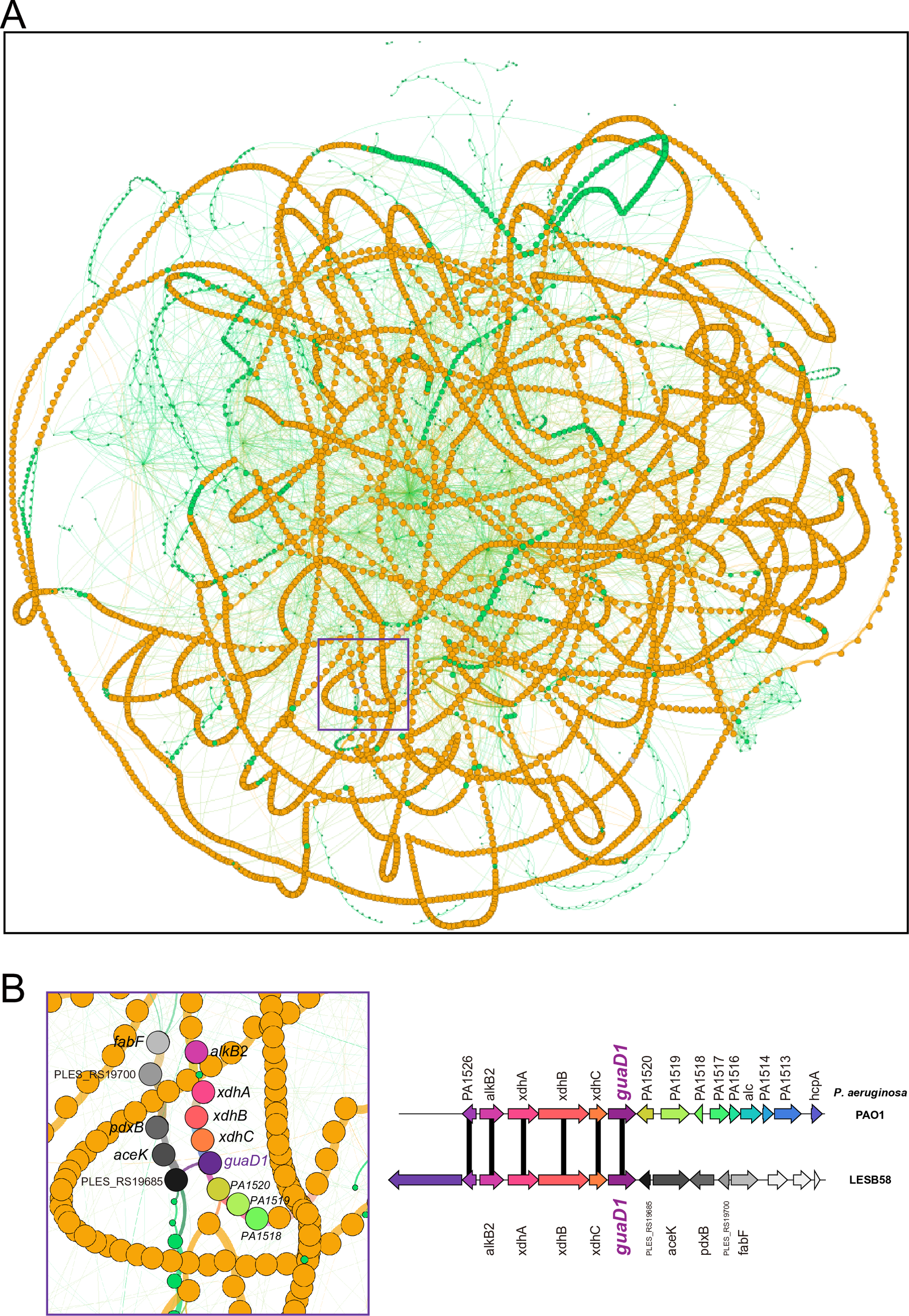

Supplement: S6 Fig — (A) Pangenome graph of 391 P. aeruginosa genomes. The frame shows a zoom of genomic region containing guaD1 and its flanking genes. (B) Two typical strains with one of two types of genomic regions flanking guaD1 in P. aeruginosa, respectively. The amplified zoom (left) and comparative analysis of genomic regions (right). The gene arrangement was indicated with identical colors. (TIF) [file pone.0315931.s006.tif]

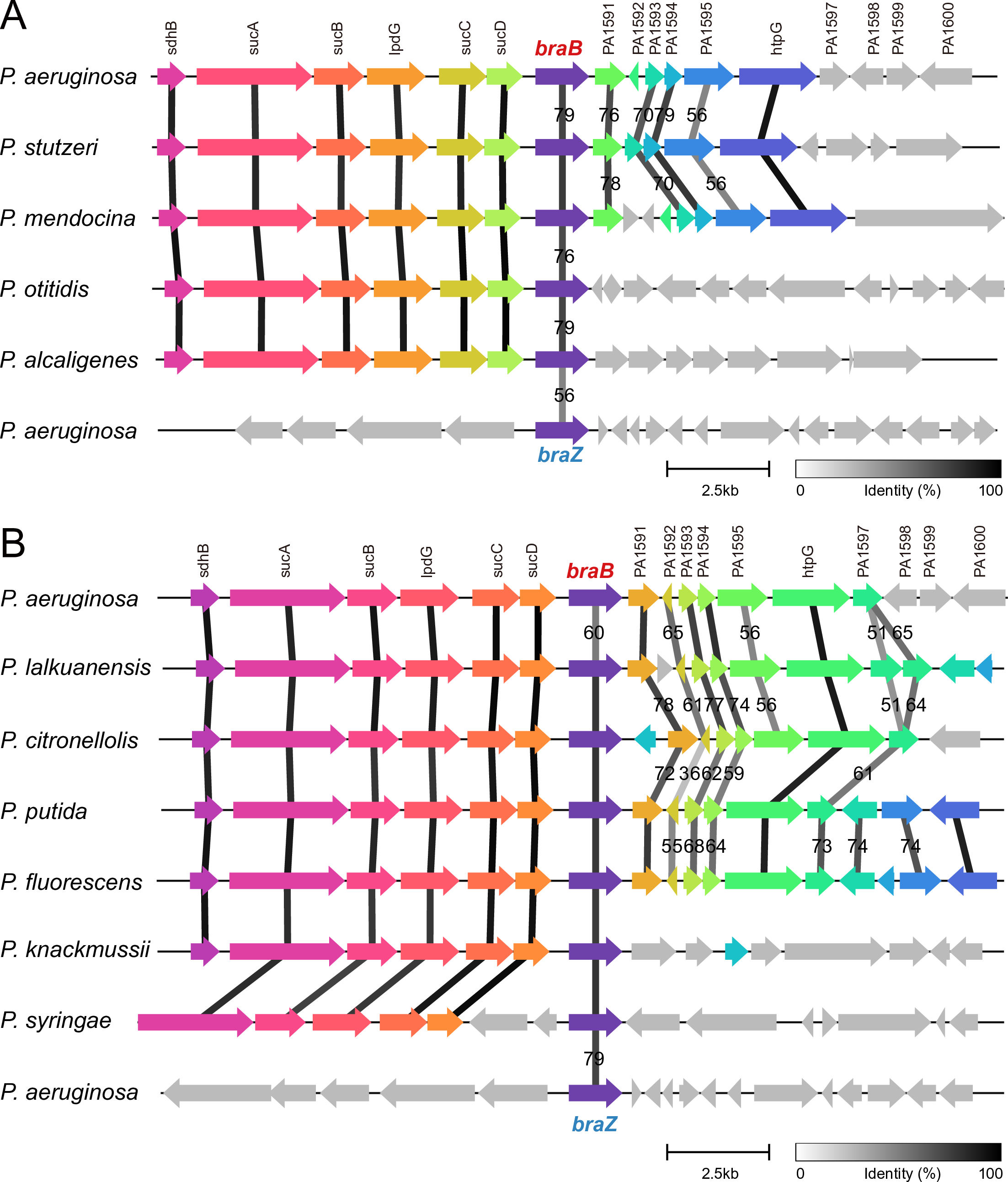

Supplement: S7 Fig — Colorful arrows and dark shading indicate the gene direction and nucleotide sequence identity of conserved regions. The nucleotide sequence identity less than 80% are indicated. The scale bar indicates the length of 2.5kb nucleotides. There was no co-linearity between genomic regions flanking transporters specific to BCAAs in each species and that of braZ. (A) It is about four species in Clade I in Fig 2A (right) with their transporters specific to BCAAs more similar to braB than braZ. (B) It is about six species in Clade II in in Fig 2A (right) with their transporters specific to BCAAs more similar to braZ than braB. (TIF) [file pone.0315931.s007.tif]

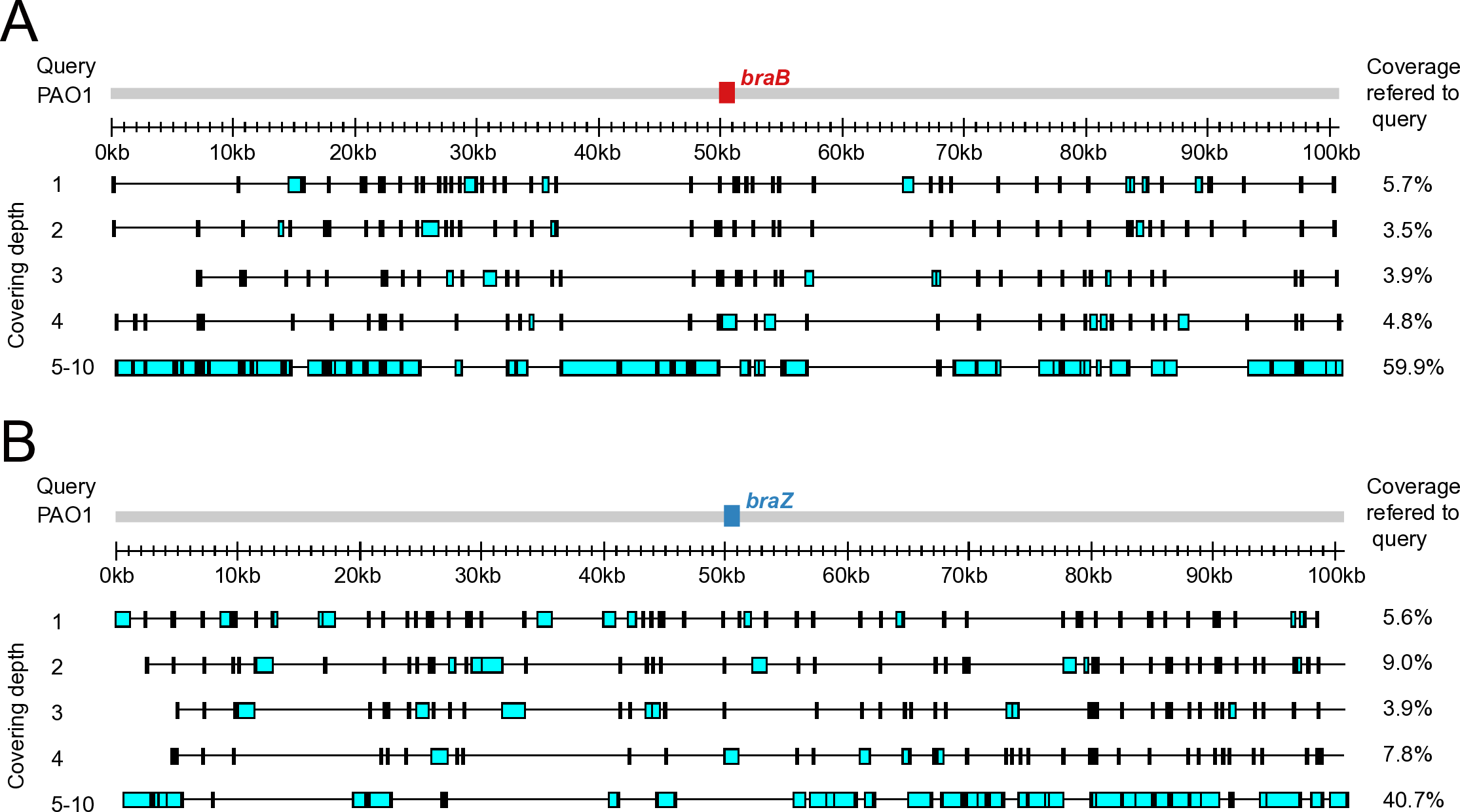

Supplement: S8 Fig — The 10 species refer to those species used for comparative analysis with PAO1 in Fig 2C. The blue filled boxes on the line represent the length and the position of matched regions in each species relative to the query in PAO1 under a given covering depth. The proportion of matched regions occupying the query sequence is estimated and indicated with coverage referred to the query. About 9kb of genomic region flanking braZ was lost in other species, while 20kb of genomic region flanking braB maintained stable in other species in Pseudomonas. (TIF) [file pone.0315931.s008.tif]

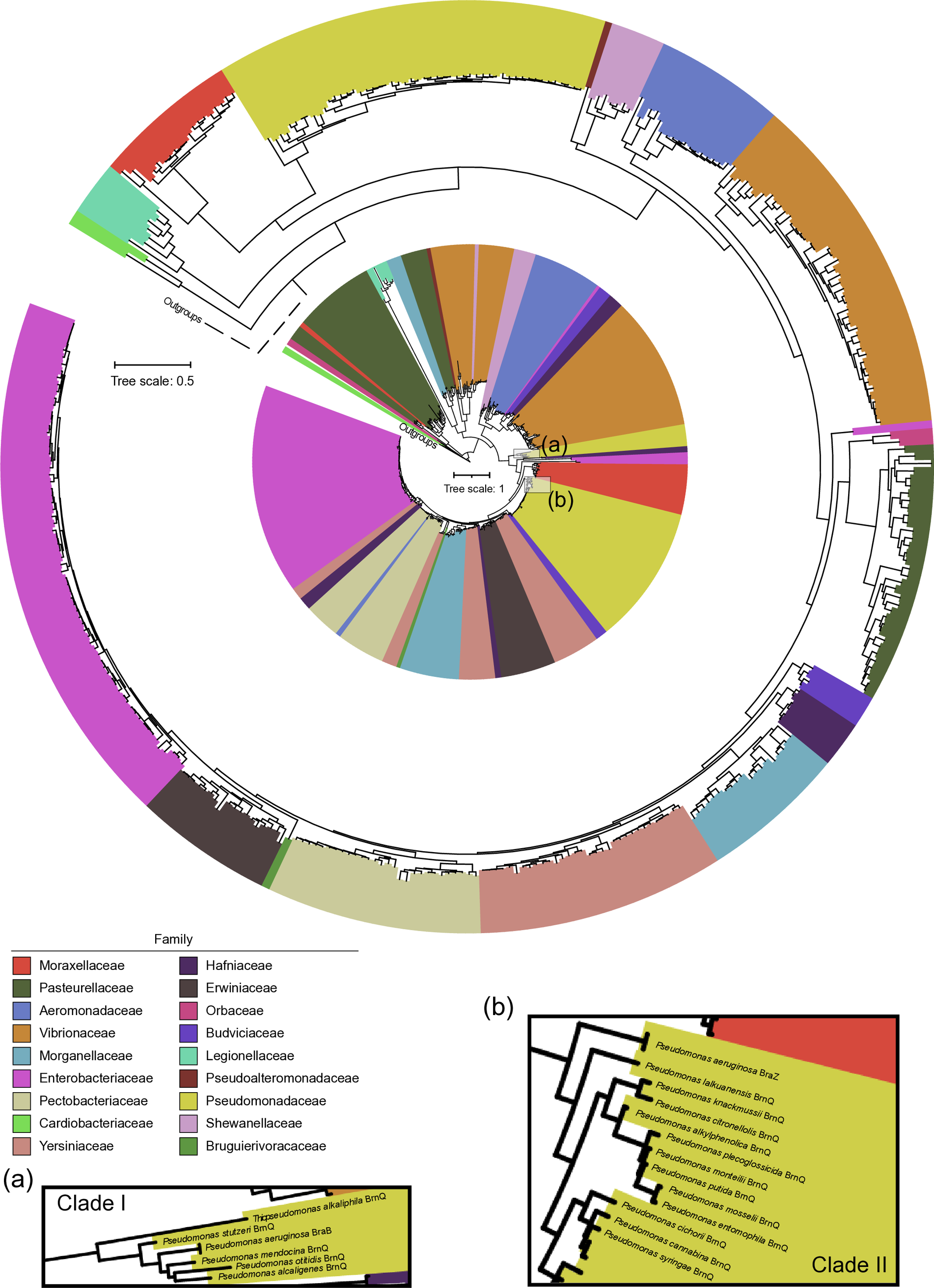

Supplement: S9 Fig — The scale bar represents 0.5 substitution per site for the species tree, as well as 1 substitution per site for the protein tree. Species adjacent to P. aeruginosa BraB or BraZ are displayed in a zoom, with (a) for Clade I and (b) for Clade II. (TIF) [file pone.0315931.s009.tif]

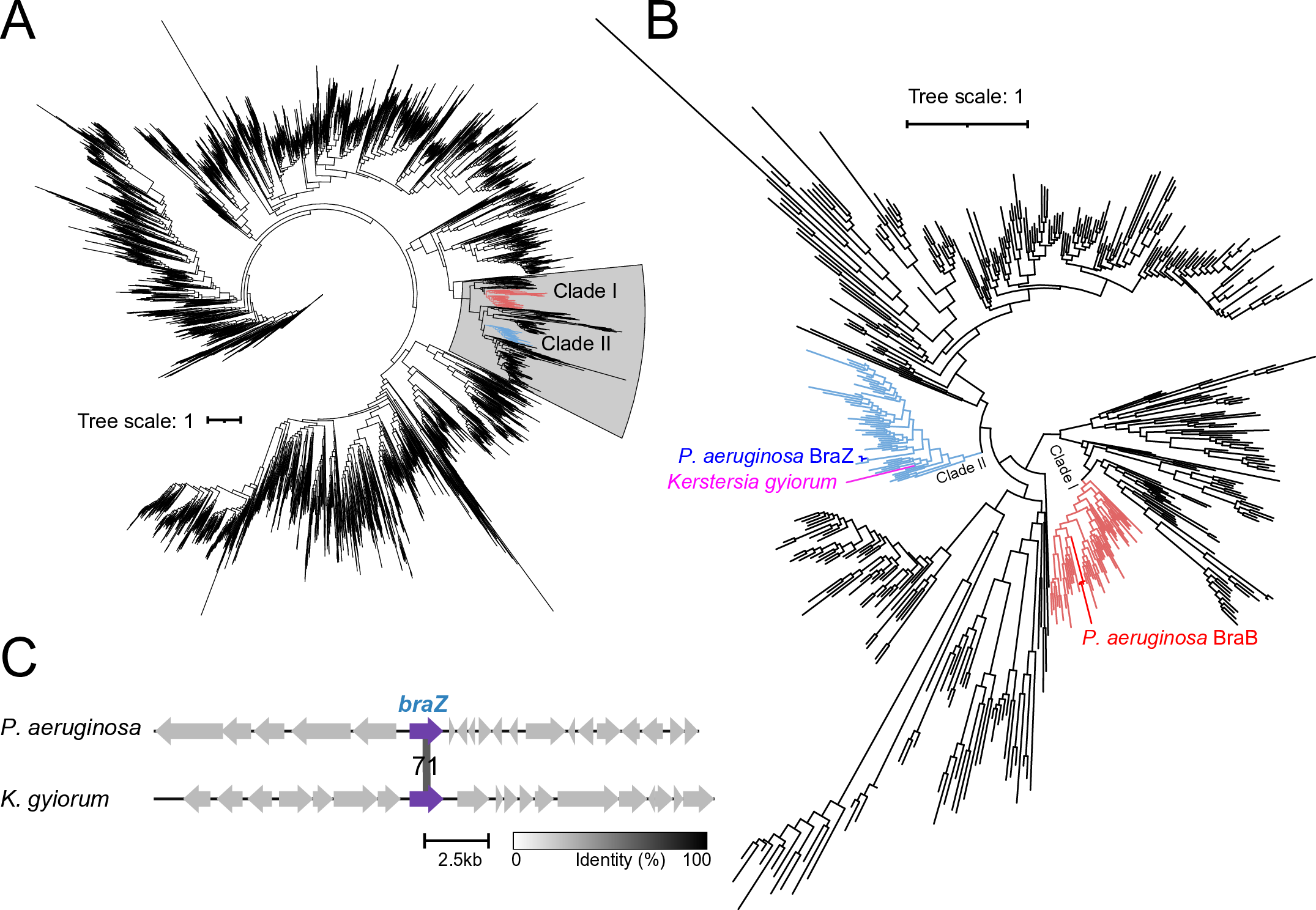

Supplement: S10 Fig — The scale bar indicates 1 substitution per site. (A) All hits in NR database were clustered by CID-HIT with 90% sequence identity as a threshold. For BarT in Pseudomonas, Clade I and Clade II were marked with light red and light blue consisting of BraB and BraZ in P. aeruginosa, respectively. The clades marked by grey boxes consist of the phylogenetic positions of BraB and BraZ, and their adjacent clades. (B) Phylogenetic tree of reconstructing the phylogenetic relationship using IQ-TREE for the protein sequences in the branch marked by the grey box in A. The representative protein sequences selected by CID-HIT were aligned with mafft and trimmed by trimAl. The resulting sequences were subject to the phylogenetic analysis using IQ-TREE. The possible evolutionary source (Keratersia gyiorum) of BraZ is highlighted with mulberry. (C) Comparative analysis of genomic region flanking braT in K. gyiorum and that of braZ in P. aeruginosa PAO1. (TIF) [file pone.0315931.s010.tif]

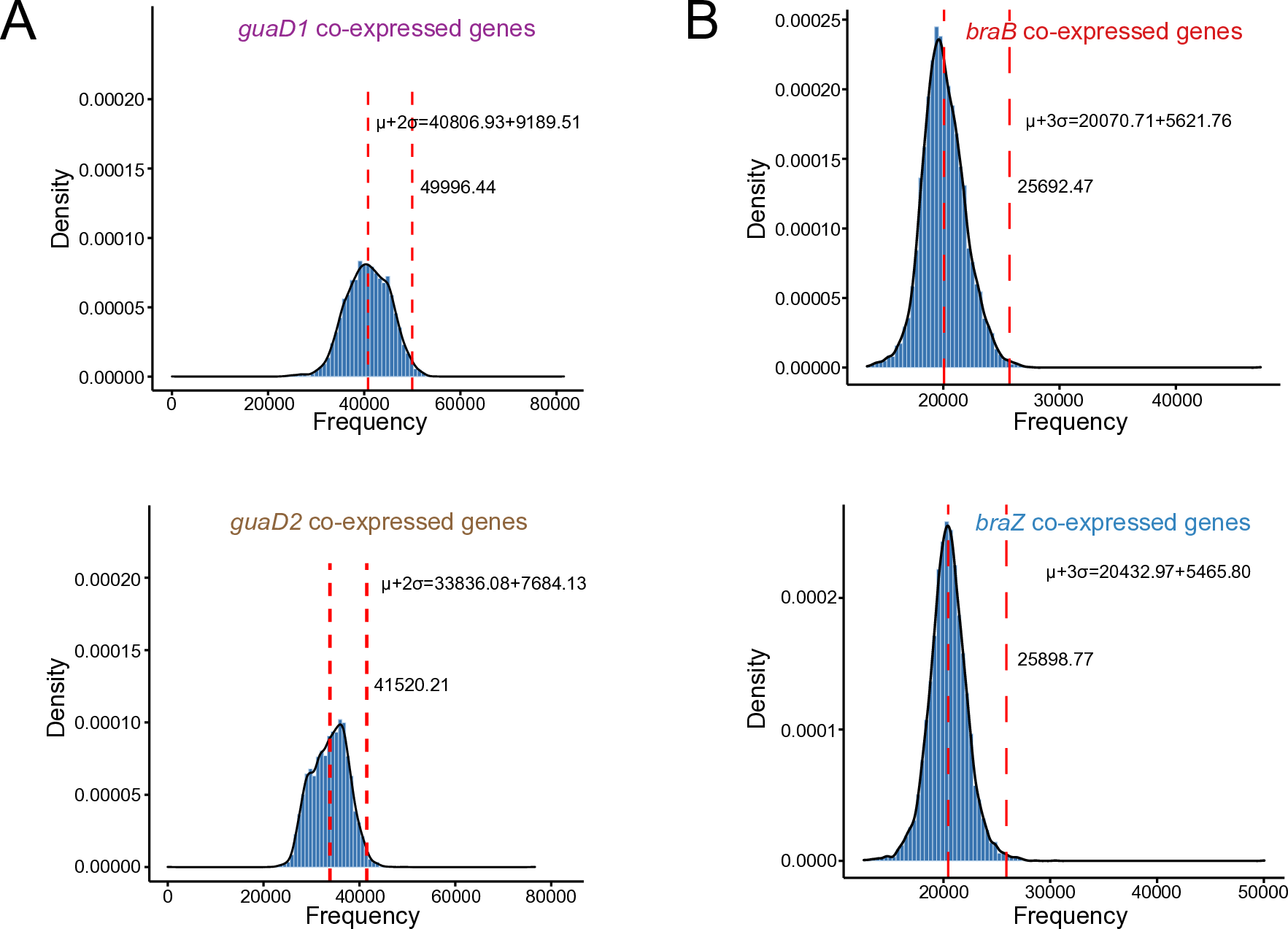

Supplement: S11 Fig — Density stands for the distribution of co-expressed genes for a given number of combinations (Frequency). Normal distribution curves were added to determine μ and σ using the ggplot2 package. Based on the 2σ principle, the value of μ+2σ was used as the threshold to identify the most significantly co-expressed genes of guaD1 and guaD2. Based on the 3σ principle, the value of μ+3σ was used as the threshold to identify the most significantly co-expressed genes braB and braZ. (TIF) [file pone.0315931.s011.tif]

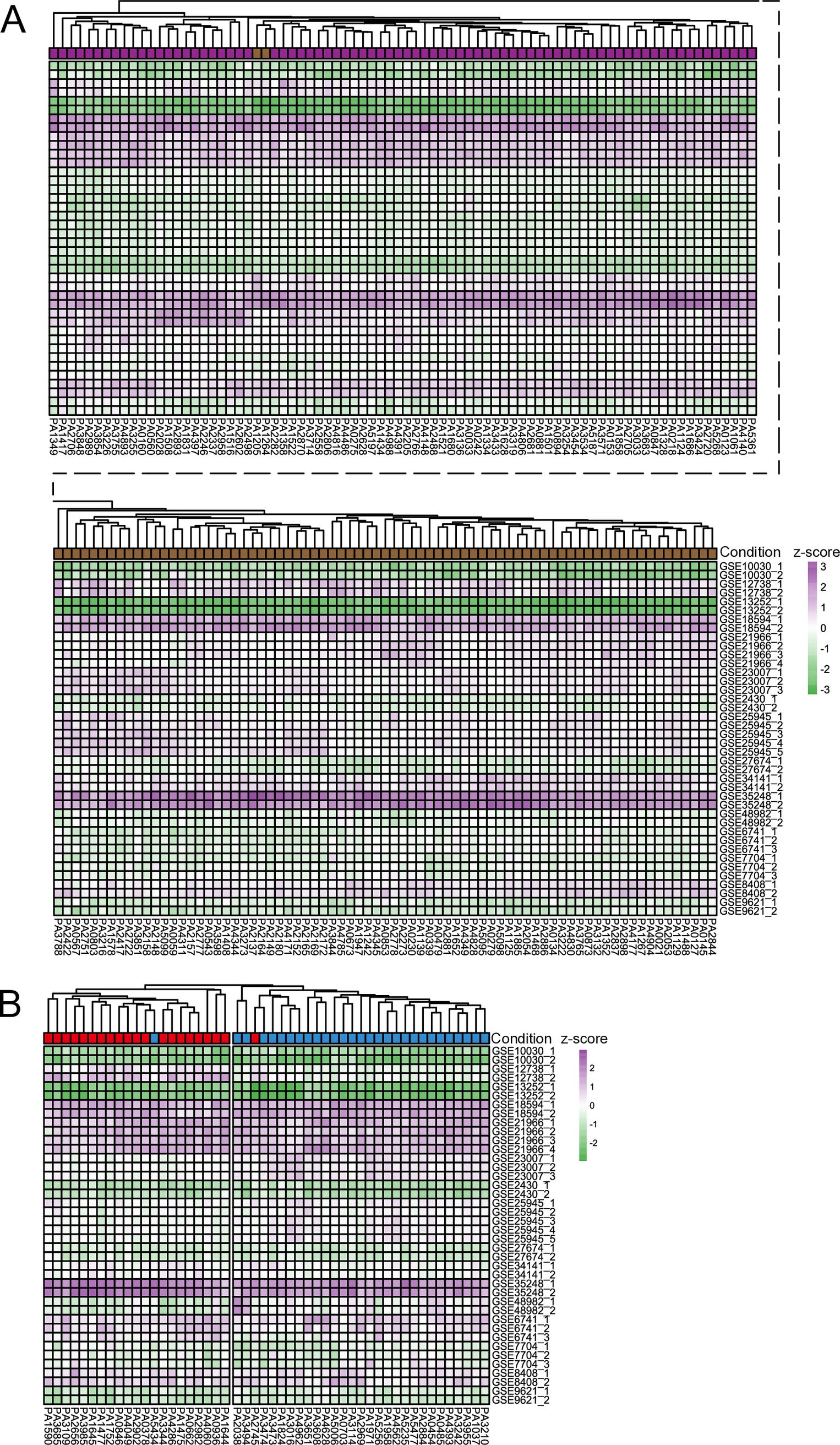

Supplement: S12 Fig — Z-scores by RMA function were used as the input. (A) Respective co-expressed genes of guaD1 and guaD2 were marked with the same colors referred to guaD1 and guaD2 in Fig 1A (center), respectively. (B) Respective co-expressed genes of braB and braZ were marked with the same colors referred to braB and braZ in Fig 1A (right), respectively. (TIF) [file pone.0315931.s012.tif]
