# Supplementary material for: Evolution of two metabolic genes involved in nucleotide and amino acid metabolism in Pseudomonas aeruginosa
Source: PLoS One. 2024 Dec 17;19(12):e0315931. doi: 10.1371/journal.pone.0315931 (PMC11651626; doi:10.1371/journal.pone.0315931)
Supplement: Attachment — Submitted filename: PONE-D-24-33220_reviewer_comments.pdf [file pone.0315931.s023.pdf]

This research article from Wu, Shi, and Liang presents an interesting look into how a guanidine deaminase and a branch chain amino acid transporter evolved in the priority pathogen *Pseudomonas aeruginosa*. By understanding how *P. aeruginosa* and in turn other bacteria, we can gain an understanding of how the bacterium maintains homeostasis of the nucleotide pool within the cell. This article presents a comprehensive analysis on the evolution and acquisition of these important genes.

The discussion and conclusions within this research are impeccable, I found myself writing down questions while reading through the results which were subsequently comprehensively answered within the discussion. I would like to thank the authors for their attention to detail within this manuscript, it was enjoyable to read.

I have some questions and very minor corrections I believe are critical to evaluate and attempt to address to improve this manuscript.

Major revisions:

I have no major revisions for this manuscript, everything is minor corrections.

Questions:

Can the authors comment on the possibility of a gene duplication events occurring in *P. aeruginosa* ancestors to acquire new genes which can then diversified and co-evolved, compared acquisition of these genes from external means. Is it more or less likely to occur via external ancestors like shown here?

Minor revisions:

General comment/ Please ensure throughout the nomenclature for genes and proteins is correct and consistent. For example, *guaD* (gene, italics) and GuaD (protein, non-italics capitalised first letter).

General comment/ I find the introduction is lacking somewhat. More information could be included about what is currently known about the function of guanidine deaminase and BCAA specific transporters, and why they would be important to *P. aeruginosa* metabolism and evolution. (i.e., What do they do and why?)

Ln 32/ "Little is known about the evolution of these two metabolic genes". I understand you are referring to *guaD* and the BCAA transporter, however you only give the name of *guaD*. Please rephrase to refer to proteins or include the BCAA transporter gene name.

Ln 57/ Citation 14 is incorrect, this is not the primary source for the WHO's classification of carbapenem resistant *P. aeruginosa* as a priority pathogen. Please update with the official citation directly from the WHO

Ln 60/ "has presented key challenges for this pathogen to adapt to environmental shifts including clinical antibiotic therapy and immune stress"

Ln89-99/ I appreciate the authors having included specific version of the databases and software utilized, could you please include the citations for Pfam and TIGRFAM, and the version for MUSCLE, Faops, trimA1, IQ-TREE, and iTOL (if they exists)

Ln103/ Please cite PANTHER

Ln110/ I trust the BLASTP was used from BLAST+ via command line. Could you please provide version and citation for this

L111/ I am unsure what this sentence is trying to say

Ln118/ Please provide a version for Clinker if it exists

Ln120/ As above, for PPanGGOLiN and Gephi

Ln126/ Please include ggplot2 version used

Ln131/ likewise with affy

Ln140/ and with eggNOG

L141/ Include version and citation for clusterProfiler

Ln142/ Was this a corrected p value or raw p value as clusterProfiler can provide both

Ln150/ I believe alongside Sood et al, this would be a more appropriate citation (10.3389/fmicb.2015.01036)

Ln164/ Is there a mislabelling in Supplementary Figure 1, I cannot distinguish the two copies of BraZ, Additionally, in these supplementary figures, genes and loci are provided but when referring to bra, or gua, you label them with protein nomenclature, see 'general comment' above.

Ln165/ "Genomic analysis of four commonly used reference isolates, namely ..."

Ln167/ should this read "flanking the two copies of the two metabolic genes, except..."

Ln 175/ is it possible to include gene identification for the 5 flanking genes either side in figure 1C like shown in Supplementary figure 6B. I believe this will enhance clarity

Ln243/ Version of CD-HIT

Ln243-250/ This is borderline methods, perhaps this could be included in methods rather than in results section. Although I understand why the authors have done it this way.

Reviewer information: Dr Samuel Wardell,

University of Otago, Department of Microbiology and Immunology

Dunedin, New Zealand
